# Interspecies Differences in the Connectivity of Ventral Striatal Components Between Humans and Macaques

**DOI:** 10.3389/fnins.2019.00623

**Published:** 2019-06-14

**Authors:** Xiaoluan Xia, Lingzhong Fan, Chen Cheng, Rong Yao, HongXia Deng, Dongqin Zhao, Haifang Li, Tianzi Jiang

**Affiliations:** ^1^College of Information and Computer, Taiyuan University of Technology, Taiyuan, China; ^2^Brainnetome Center, Institute of Automation, Chinese Academy of Sciences, Beijing, China; ^3^University of Chinese Academy of Sciences, Beijing, China; ^4^Experimental Teaching Center, Shanxi University of Finance and Economics, Taiyuan, China; ^5^National Laboratory of Pattern Recognition, Institute of Automation, Chinese Academy of Sciences, Beijing, China; ^6^Queensland Brain Institute, The University of Queensland, Brisbane, QLD, Australia

**Keywords:** ventral striatum, nucleus accumbens, comparative neuroimaging, connectivity fingerprint, parcellation

## Abstract

Although the evolutionarily conserved functions of the ventral striatal components have been used as *a priori* knowledge for further study, whether these functions are conserved between species remains unclear. In particular, whether macroscopic connectivity supports this given the disproportionate volumetric differences between species in the brain regions that project to the ventral striatum, including the prefrontal and limbic areas, has not been established In this study, the human and macaque striatum was first tractographically parcellated to define the ventral striatum and its two subregions, the nucleus accumbens (Acb)-like and the neurochemically unique domains of the Acb and putamen (NUDAPs)-like divisions. Our results revealed a similar topographical distribution of the connectivity-based ventral striatal components in the two primate brains. Successively, a set of targets was extracted to construct a connectivity fingerprint to characterize these parcellation results, enabling cross-species comparisons. Our results indicated that the connectivity fingerprints of the ventral striatum-like divisions were dissimilar in the two species. We localized this difference to specific targets to analyze possible interspecies functional modifications. Our results also revealed interspecies-convergent connectivity ratio fingerprints of the target group to these two ventral striatum-like subregions. This convergence may suggest synchronous connectional changes of these ventral striatal components during primate evolution.

## Introduction

The primate ventral striatum is comprised of the nucleus accumbens (Acb), the broad continuity between the caudate nucleus and the putamen ventral to the rostral internal capsule, the olfactory tubercle (Heimer et al., 1999), and the medial caudate nucleus from a connectional perspective (Haber and McFarland, 1999). This specialized part of the striatal complex integrates signals from both the prefrontal and limbic areas of the brain and plays a prominent role in refining action selection by augmenting the efficiency and vigor of either appetitively or aversively motivated behaviors (Floresco, 2015). Furthermore, it has been an important target for deep brain stimulation and novel biological therapies for anxiety and obsessive-compulsive disorders, including depression and addiction (for reviews, see Sturm et al., 2003; Blomstedt et al., 2013; Gelfand and Kaplitt, 2013).

Given that the ventral striatum (or more specifically, the Acb core) extends slightly into the caudate nucleus and putamen, reaching the medial caudate nucleus border (Brauer et al., 2000), the dorsal boundary of this region has yet to be well-defined histochemically. In contrast, it has been delineated by the specifications suggested by more modern anatomical analyses (for details, see Supplementary Material, Section “Anatomical Boundaries of the Acb”). In addition, our group found that this region was not well-defined by magnetic resonance imaging (MRI), at least as provided by Calabrese et al. (2015) on a high-resolution T2-weighted image at a 0.15 mm isotropic resolution, due to a lack of distinct signal intensities. Complicating things further, no definitive conclusions about the correspondence between the microstructural and connectional boundaries of this region exist. In fact, a ventral striatum-like subregion defined by connectivity-based parcellations of the human striatum in previous studies showed a significant extension into the dorsal striatum (Tziortzi et al., 2013; Janssen et al., 2015; Fan et al., 2016). Thus, the histochemically and anatomically defined ventral striatum area may not be a connection unit, i.e., an area consisting of voxels having similar connectivity features, and thus may not be the optimal selection for the region of interest for connection analyses. In short, a relatively accurate connectivity-defined ventral striatum area is needed to meet the demands of ventral striatum-related neuroimaging connectivity analyses.

The evolutionarily conserved functions of the brain regions in the ventral striatum were suggested (Izawa et al., 2003; Calipari et al., 2012; Daniel and Pollmann, 2014) and used as *a priori* knowledge for further studies (Neubert et al., 2015; Heilbronner et al., 2016). Although many microstructural features and intrinsic axonal projection patterns of this nucleus have been qualitatively described across species and found to be similar, supporting the hypothesis that the functions are evolutionarily conserved (Basar et al., 2010; Salgado and Kaplitt, 2015; Volkow and Morales, 2015), conservation of their macroscopic connectivity architecture has yet to be identified. In light of the concept that nothing defines the functions of a brain region better than its connections (Knosche and Tittgemeyer, 2011; Cloutman and Lambon Ralph, 2012; Glickfeld et al., 2013), calculating and comparing the connectivity patterns of brain regions across species is of crucial importance for testing whether this hypothesis can be supported by macroscopic connectivity. In addition, genetic and developmental heterochronic shifts of the human striatum (Sousa et al., 2017) have brought changes in the direction of trait variation on a macro-evolutionary scale, such as a significantly smaller volume than predicted based on primate scaling trends (Barger et al., 2014). Interspecies volumetric abnormal changes also exist in some brain regions that are strongly connected to the ventral striatum, including the evolutionary expansions of the human prefrontal cortex, amygdala (Amyg), and the hippocampus (Hipp) (Somel et al., 2011; Barger et al., 2014; Carlén, 2017; Smaers et al., 2017). All of these volumetric abnormal changes may be accompanied by unique modifications of their connectivities with the ventral striatal components.

Comparative MRI allows us to make large-scale (large sample, multiple species, and modalities) comparative analyses and is one of the few techniques that can truly bridge the gap between species by using identical non-invasive data acquisition schemes and data handling (Thiebaut de Schotten et al., 2018). Diffusion MRI tractography is useful for discovering meaningful patterns of brain evolution and has become increasingly popular in primate comparative neuroscience (Mars et al., 2016b). For example, it has been used to describe differences between humans and non-human primates in the specific white matter pathways involved in language (Rilling et al., 2008, 2011) along with many other white matter fibers (Zhang et al., 2013; Hecht et al., 2015). In addition, Passingham et al. (2002) proposed the connectivity fingerprint as a way to summarize the important connections of a single brain area (termed the seed) with a select set of other areas (termed the targets) and indicated it was a physiologically unique identifier of the seed area. The connectivity fingerprint and its variant, the connectivity blueprint, can be used to establish a common space in which a brain area’s connectivity patterns, as characterized in different brains, can be aligned and quantified across species (Mars et al., 2018a, b). Such fingerprint-defined common space approaches more fully exploit the possibilities offered by neuroimaging techniques and have been used in many recent comparative MRI studies (Mars et al., 2012, 2016a, Mars et al., 2018b; Neubert et al., 2015).

The goal of this study was to compare the macroscopic connectivity of the ventral striatum and its subregions between humans and macaques. To address this issue, we first recursively parcellated the striatum, based on probabilistic diffusion tractography, to define the connection units of the ventral striatal components for each species. Then, we selected a set of target areas to construct the fingerprint framework for cross-species comparisons. Using these target areas, we characterized the anatomical connectivity patterns of the ventral striatum and its subregions. Finally, we evaluated the similarity of the corresponding connectivity fingerprints between the two species and detected the interspecies single connectivity differences to analyze possible interspecies functional modifications.

## Materials and Methods

### Subjects, MRI Data Acquisition, and Preprocessing

A human MRI dataset (40 subjects; age: 22–35; 22 males) was extracted from the Human Connectome Project (HCP; Van Essen et al., 2013) because of the relatively high registration accuracy of the Acb region in our earlier study (Xia et al., 2017). The data included high-quality structural images (0.7 mm isotropic resolution) and diffusion images acquired using a multi-shell approach at a 1.25 mm isotropic resolution (for more details, see Sotiropoulos et al., 2013; Ugurbil et al., 2013).

A macaque MRI dataset consisting of eight adult rhesus macaque brain specimens (i.e., *ex vivo*; age: 4–23 years; two males; for details, see Supplementary Material, Section “Preparation of the Specimens and MRI Data Acquisition”) was used in this study. These monkeys were obtained from the Kunming Institute of Zoology, Chinese Academy of Sciences (CAS), and were judged by veterinarians to be appropriate subjects for euthanasia due to the presence of serious physical diseases. All brain specimens were obtained at necropsy immediately following euthanasia due to reasons not related to the study and acquired following protocols approved by the National Animal Research Authority of China. The details of the preparation of the brain specimens are provided in Supplementary Material, Section “Preparation of the Specimens and MRI Data Acquisition.” Immediately prior to imaging, the specimens were transferred to an MRI-compatible holder, bundled with medical gauze, and immersed in Fomblin (Solvay, Brussels, Belgium) to prevent dehydration and susceptibility to artifacts. The MRI data were acquired on a 9.4T horizontal animal MRI system (Bruker Biospec 94/30 USR) with Paravision 6.0.1 (for more parameters, see Supplementary Material, Section “Preparation of the Specimens and MRI Data Acquisition”). High-resolution diffusion MRI data (voxel sizes = 0.6 mm × 0.6 mm × 0.6577 mm) included 60 diffusion weighted images with the *b*-value = 1000 s/mm^2^ and four non-diffusion weighted images. The data quality controls used on these macaque diffusion images are described in Supplementary Material, Section “MRI Data Quality Checking.” All experimental procedures were performed in strict accordance with the recommendations in the National Institutes of Health Guide for the Care and Use of Laboratory Animals. All of the animals were handled according to the protocol (#IA-2016-05) approved by the Animal Care and Use Committee of the Institute of Automation, CAS.

We used these datasets to define the connection units of the ventral striatum and its subregions and then characterized them using macroscopic anatomical connectivity. The sample size in this study is comparable to that of many earlier parcellation studies (Mars et al., 2012; Liu et al., 2013; Zhuo et al., 2016). The human MRI data had been processed and used in our earlier study (Xia et al., 2017). To mitigate the influence of certain preprocessing steps on the cross-species comparisons, the preprocessing steps for the macaque MRI data were almost identical to those used for the human MRI data (for detailed preprocessing procedures, see Supplementary Material, Section “MRI Data Preprocessing”).

### Defining the Ventral Striatal Connection Units

Unlike the ventral striatum, its parent structure, the striatum, can be clearly delineated using high-resolution structural imaging. Therefore, we extracted the tail-cut striatum to perform a tractography-based parcellation to define the connectivity-based ventral striatum and its subregions, i.e., the ventral striatal connection units. This procedure (for details, see Supplementary Material, Section “Tractography-Based Parcellation Procedure”) is similar to the automatic tractography-based parcellation pipeline program (ATPP; **RRID:SCR_014815**; Li et al., 2017), which was developed to realize parcellation using automatic processing and massive parallel computing. It can be described briefly as follows: FMRIB’s FIRST was used to extract each individual’s striatum. After manual correction, tail-cut striatum was transformed from structural to diffusion space using a 6 degrees of freedom (DOF) FSL FLIRT boundary-based registration algorithm (BBR; Greve and Fischl, 2009). The whole-brain probabilistic tractography was implemented for each voxel in the striatum. Note that the probability counts were corrected by the length of the pathway to compensate for the distance-dependent bias (Tomassini et al., 2007). Then, the cross-correlation matrix was constructed (Johansen-Berg et al., 2004) to perform a cluster analysis. Voxels having similar connectivity architecture were grouped together to constitute subregions. All the individual parcellation results were co-registered to the structural space using the six DOF FSL FLIRT BBR algorithm and then transformed into Montreal Neurological Institute (MNI) space (for humans) or MNI monkey space (for macaques; Frey et al., 2011) using the state-of-the-art ANTs’ diffeomorphic transformation model (Avants et al., 2008) to generate the group-level parcellation results, including the probability map for each subregion and the maximum probability map (MPM) for the tail-cut striatum. The probability map was calculated using a group of locationally corresponding individual clusters; it thus reflects the inter-individual variability of the respective area (Caspers et al., 2008). The MPM was generated at the group level by assigning each voxel to the area in which it was most likely to be located (Eickhoff et al., 2006). The MPM allows for the retention of quantitative information about inter-subject variability.

Unlike previous parcellation procedures (Liu et al., 2013; Fan et al., 2016; Li et al., 2017; Xia et al., 2017), the connectivity probability maps of voxels in the striatum in this study were not down-sampled to avoid the loss of connectivity details. This led the tractography-based parcellation of the striatum to be time-consuming, especially when the cluster number was preset at a high value (e.g., >10). In addition, large amounts of time and resources would have been spent on parcellating the dorsal striatum, which was not the focus of the present study. For these reasons, a recursive parcellation procedure was employed for the relatively large subcortical structure of the striatum, as follows. A modest maximum of 8 clusters for macaques and 10 clusters for humans in the 1st parcellation was preset to generate the ventral striatum connection unit. Then, we parcellated the ventral striatum connection unit to generate ventral striatal subregions. In 1st parcellation, the average Cramer’s V (CV) was used to judge the consistency of the spatial distribution of these subregions among individuals like earlier studies (Fan et al., 2016; Li et al., 2017). Specifically, subjects were randomly divided into two groups and the MPMs of such groups were evaluated using CV. This step was repeated 100 times to compute the average consistency and obtain the optimal solution, which was defined by the peak of the average CV and indicated a better split-half reproducibility than the surrounding solutions. Furthermore, a subregion located in the ventral part of the striatum was extracted from the optimal solution to perform a subsequent recursive parcellation. The latter, similar to a previously described procedure (Beckmann et al., 2009; Neubert et al., 2015), involved dividing the region into two smaller subdivisions and, thereafter, further dividing the resulting regions through many steps. The anatomically-defined *a priori* location information of the ventral striatum and its major components (e.g., the Acb) was used as a reference to decide which subregions should be chosen as the connection units of the ventral striatum and its major components.

### Definition of the Comparative Framework

The connection units that had corresponding locations in the two species were treated as seeds for cross-species comparisons. The connectivity fingerprint was chosen as a comparative characteristic (Mars et al., 2016b) to detect the macroscopic connectivity conservatism of these seeds. The fingerprint framework was constructed using a set of targets with the following two criteria: (1) having strong connection with the human or macaque ventral striatum connection unit; (2) having evidence-based homologous areas between the two species. In addition, all the targets were extracted from existing atlases, as will now be described. A combination of human atlases mentioned in our earlier study (Xia et al., 2017), the Harvard-Oxford probabilistic atlases covering both cortical and subcortical areas (Desikan et al., 2006), the Oxford thalamic connectivity atlas (Behrens et al., 2003), and the dopaminergic midbrain probabilistic atlas (Murty et al., 2014), were chosen for this investigation. All the above-mentioned atlases were thresholded at 50% and the final combined atlas included 61 brain regions for each hemisphere. In contrast, the macaque targets were extracted from a histological brain atlas (Paxinos et al., 2009; Calabrese et al., 2015). The small subdivisions in this atlas were combined into their parent structure to approximately match the accuracy of the above-described human combined atlas.

Earlier studies calculated relative connectivity strength (RCS) by normalizing the data to the maximum regional connectivity strength in the brain to build connectivity fingerprints for cross-species comparison (Mars et al., 2012, 2016b; Neubert et al., 2015). However, the RCS calculated in different primate brains or even different brains within a species may be affected by inconsistent normalization factors, e.g., maximum regional connectivity strength. In the current study, however, since probabilistic tractography was calculated for each seed by sampling the same 50,000 streamlines to estimate the connectivity probability for both types of primate brains, we calculated the RCS by normalizing the data to the whole-brain mean connectivity strength (another normalization factor) to build ‘connectivity strength fingerprints’ of the ventral striatum for cross-species comparison. Nevertheless, the RCS cannot avoid the effect of inconsistent normalization factors when using different MRI acquisition parameters, different data processing methods, and inconsistent normalization factors. For this reason, we used another connectivity characteristic, i.e., connectivity ratio (CR), to characterize the connectivity of the ventral striatum subregions. Given a target identified above according to the two criteria, we first calculated the anatomical RCS between this target and each ventral striatal subregion. Then, we defined the connectivity ratio of one of the ventral striatal subregions as:

$$\mathrm{CR}\,\left(\mathrm{target},\mathrm{seed}\,\left(i\right)\right)=\frac{\mathrm{RCS}\,\left(\mathrm{target},\mathrm{seed}\,\left(i\right)\right)}{\sum_{j=1}^{n}\mathrm{RCS}\,\left(\mathrm{target},\mathrm{seed}\,\left(j\right)\right)}$$

Where, seed(*i*) is one of the n ventral striatal subregions; RCS(target, seed(*i*)) is the RCS between the given target and seed(*i*); CR(target, seed(*i*)) is the connectivity ratio of seed(*i*).

The CR(target, seed(*i*)) has values in the interval [0, 1], and high values indicate that the target tends to connect with the seed(*i*) when compared with the other ventral striatal subregions. Therefore, the connectivity ratio fingerprint reflects the contrast in the general connectivity trend across the ventral striatal subregions. We used the CR fingerprint to detect possible interspecies differences in the connectivity trends for the ventral striatal components.

### Characterization of the Ventral Striatal Components

Employing a similar calculation procedure to that of our earlier study (Xia et al., 2017), pairs of seeds of the two species in MNI space or MNI monkey space were brought back to the subjects’ diffusion space. For each seed, the whole-brain connectivity probability map was generated in individual diffusion space, and thresholded at *p* > 0.04% (i.e., 20 out of 50,000 streamlines fibers) to reduce false positive connections as was done in earlier studies (Fan et al., 2016; Li et al., 2017; Xia et al., 2017). Subsequently, all individual connectivity probability maps were transformed into MNI space or MNI monkey space to generate a group-averaged connectivity probability map of the seed. In addition, as described in previous studies (Fan et al., 2016; Li et al., 2017; Xia et al., 2017), we generated the binary images of the individual connectivity probability maps. Then, we generated a group-averaged map using these binary images and thresholded it at *p* > 50% to get a binary image, i.e., the common fiber tract map. The generation of the common fiber tract map was, in effect, analogous to a one-sample *t*-test for determining the voxels that had significant connectivity with the seed. We used the common fiber tract map to mask the group-averaged connectivity probability map to further reduce false positive connections and the effects of individual differences.

The anatomical RCS between the ventral striatum connection unit and each area in the selected atlas was calculated using the group-averaged connectivity probability map. A set of brain areas that presented strong connections with either the human or macaque ventral striatum connection unit (for details, see Supplementary Material, Section “Definition of the Criteria for the Target Areas”) was extracted and used to represent the candidate targets. Subsequently, previous studies were consulted to determine whether obvious interspecies differences existed in each area. The areas lacking such an association were extracted from the atlas and considered members of the target group. Based on this target group, in both species, we characterized the ventral striatum-like division using the RCS fingerprint to reflect the connectivity profile of the ventral striatum and characterized the subregions of the ventral striatum-like division using the CR fingerprint to reflect the gross connectivity trend of these targets to these ventral striatal components.

### Cross-Species Comparisons

As in recent comparative neuroimaging studies (for a review, see Mars et al., 2016b), the connectivity fingerprints were employed in the present study to investigate the relationship between the variances in the organization of different brains. We defined the null hypothesis as “the corresponding connectivity fingerprints of the ventral striatal components are consistent between the two species.” We generated the group-averaged connectivity fingerprints for the human and macaque ventral striatum and its subregions and calculated the observed cosine similarity between the corresponding connectivity fingerprints. Subsequently, we performed the following procedure 1000 times to create the permutation distribution: (1) Two groups (human and macaque groups) of connectivity fingerprints were merged and then randomly divided into two groups (their sample sizes were kept constant); (2) We generated the group-averaged fingerprints and calculated their cosine similarity. Finally, the test criterion was the 5% significance level. If the null hypothesis was true, the two groups of the connectivity fingerprints would have the same distribution and the observed cosine similarity would not be a rare cosine similarity value in the permutation distribution. Subsequently, we further located the connectivity differences between dissimilar fingerprints to specific targets by performing single connection analyses. An independent two-sample *t*-test at the 5% significance level was used to determine any significant interspecies connectivity difference in this procedure.

## Results

### Connectivity-Based Region of the Ventral Striatum

**FIGURE 1:**
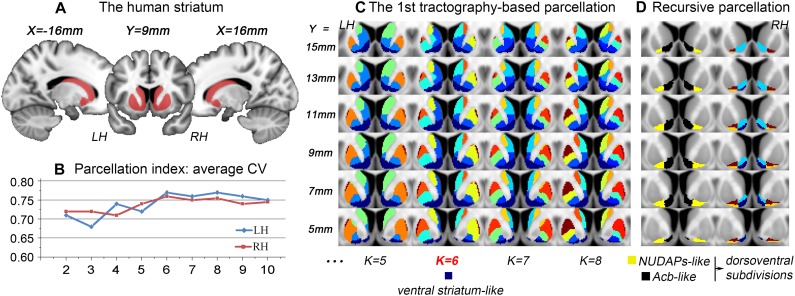
Tractography-based parcellation of the human striatum. **(A)** The tail-cut human striatum was chosen as the seed mask. All the coordinates are shown in MNI space (similarly hereafter). **(B)** The parcellation index of the average CV indicates that the six-cluster solution is the optimal data description. **(C)** The results of the 1st parcellation of the human striatum are displayed in a multi-slice presentation. The dark blue cluster in the six-cluster solution was named the ventral striatum-like division and extracted for subsequent recursive parcellations. Finally, the resulting parcels are illustrated in **(D)**; the black and yellow clusters were named the Acb-like and NUDAPs-like divisions, respectively. The two clusters both presented a dorsoventral subdivision. LH, left hemisphere; RH, right hemisphere.

**FIGURE 2:**
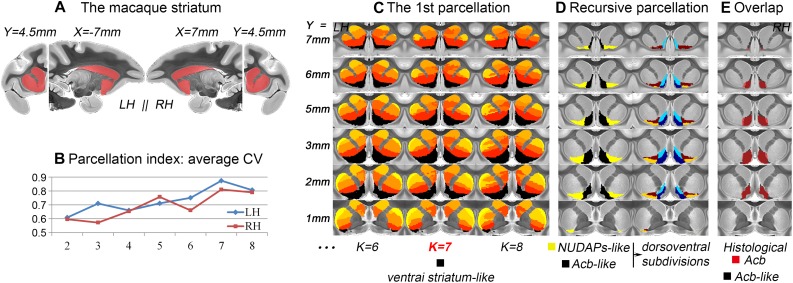
Tractography-based parcellation of the macaque striatum. **(A)** The tail-cut macaque striatum was chosen as the seed mask. **(B)** The parcellation index of the average CV indicates that the 7-cluster solution is the optimal data description. **(C)** The results of the 1st parcellation of the macaque striatum. The black cluster in the 7-cluster solution was named the ventral striatum-like division and extracted for subsequent recursive parcellations. **(D)** The parcellation results of the ventral striatum-like cluster. **(E)** The resulting macaque Acb-like division presents a high overlap with the histological Acb.

After the 1st tractography-based parcellation of the striatum (Figures 1A, 2A), a six-cluster solution in humans (Figure 1B) and a seven-cluster solution in macaques (Figure 2B) presented the best CV-based data description and were therefore considered the optimal solutions for the two species. In addition, the subregion located in the anteroventral part of the human and macaque striatum in the optimal solution overlapped considerably with those in the surrounding solutions (see the dark blue clusters in Figure 1C and the black clusters in Figure 2C), suggesting that it may represent a stable region from a connectional perspective. As both these striatal subregions had a good overlap with the anatomically-defined primate ventral striatum and since both presented medio-lateral subdivisions, but not a further dorsoventral subdivision (Figures 1D, 2D), they were named the ventral striatum-like division. In contrast, the human ventral striatum-like division generated in the current study was smaller than the corresponding subregion generated in previous connectivity-based parcellations of the human striatum (Tziortzi et al., 2013; Janssen et al., 2015; Fan et al., 2016). Furthermore, given that an obvious extension of the ventral striatum-like division into the anatomically-defined dorsal striatum was not observed, this region was considered to be a more accurate definition of the ventral striatum from a connectivity perspective.

### Two Subregions of the Ventral Striatum

The ventral striatum is often also referred to as the Acb in anatomy and relatively low-resolution neuroimaging (Heimer et al., 1999; Fan et al., 2016). Unlike previous studies, the human and macaque ventral striatum-like divisions were further parcellated in the present research into medial and lateral parts. The medial subregion extracted from the MPM in macaques presented a high Dice coefficient (LH: 84.4%; RH: 83.6%; Figure 2E) with the histological Acb region of the rhesus macaque brain (Paxinos et al., 2009; Calabrese et al., 2015). In contrast, the lateral subregion extracted from the MPM appeared similar to the histologically-described region of the “neurochemically unique domains of the accumbens and putamen (NUDAPs)” in primates. This brain region comprises many patch-like areas located in the ventral border of the Acb and the ventral one-third of the putamen, and it stands out in the [^3^H]DAMGO pattern, the distribution of the kappa opioid, and the D1-like dopamine receptors (Voorn et al., 1996; see Supplementary Figure S3). Furthermore, both the ventral striatum-like subregions presented dorsoventral subdivisions, although further medial or lateral subdivisions were not observed in the two species (Figures 1D, 2D). This suggests that, after two recursive parcellations of the ventral striatum-like division, only one sagittal surface could be found to divide this region into a medial subregion and a lateral subregion. Therefore, we considered the medial and lateral subregions of the ventral striatum-like division as the Acb and NUDAPs connection units, respectively, and named them the Acb-like and NUDAPs-like divisions (Figures 1D, 2D). The similar recursive parcellation results between the two species enabled the extraction of pairs of connection units corresponding in location, including the ventral striatum-like, Acb-like, and NUDAPs-like divisions, as the seeds for subsequent cross-species comparisons.

### Characterization and Comparison of the Ventral Striatum-Like Divisions

Fourteen targets, which presented strong connectivities with the ventral striatum-like divisions in both species, were used to construct the fingerprint framework. Given the different nomenclature across atlases, both the full name and abbreviation of these targets were changed and described as follows: area 10, located in the medial and ventral frontal pole; area 13, located in the posterior part of the medial orbital gyrus; area 14, located in the ventral prelimbic area; area 25, located in the infralimbic area; and area 32, located in the dorsal prelimbic area, insula cortex (INS), temporal pole (TP), caudate nucleus (Ca), putamen (Pu), pallidum (Pa), hippocampus (Hipp), amygdala (Amyg), mediodorsal (MD) part of the thalamus, and midbrain (MidB). The areas located in the lateral orbitofrontal cortex were eliminated from the target group considering the substantial interspecies differences reported in previous studies (Petrides et al., 2012; Neubert et al., 2015).

**FIGURE 3:**
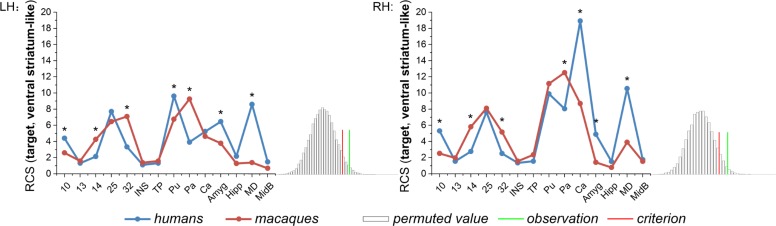
Comparison between the human and macaque ventral striatum-like division using the RCS fingerprints. Specifically, 14 corresponding areas in the two species were chosen as the target group. The RCS fingerprint of the ventral striatum-like division in humans differs greatly from that of macaques. The subsequent permutation test further showed the observed cosine similarity between the two fingerprints was higher than the calculated test criterion at the 5% significance level in the right tail. Finally, the single connection between each target and the ventral striatum-like division was calculated in individuals, whereas the connectivity difference was tested using an independent two-sample *t*-test (^*^p < 0.01).

The anatomical RCS fingerprints of the human and macaque ventral striatum-like division were calculated and compared (Figure 3). Given that the observation was a rare cosine similarity value in permutation distribution, as can be seen in the right tail of the histogram, the observed cosine similarity was higher than the calculated test criterion at the 5% significance level, the null hypothesis was rejected and the two fingerprints were considered to differ. More specifically, in the subsequent single connection analysis, the ventral striatum-like division in humans was found to present both significant stronger connectivities with area 10, Pu (only in LH), Ca (only in RH), Amyg, and MD, and significant weaker connectivities with areas 14, 32, and Pa, than those of macaques. Furthermore, significant interspecies connectivity differences were not found for the targets of the areas 13, 25, INS, TP, MidB, Ca (only in LH), and Pu (only in RH).

### Characterization and Comparison of the Ventral Striatum-Like Subregions

**FIGURE 4:**
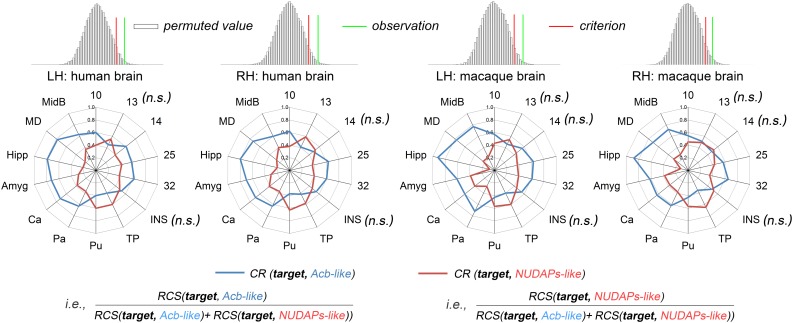
Comparison between the Acb-like and NUDAPs-like divisions using the CR fingerprints. The CR was defined as the ratio between the two RCSs. The regions present distinct CR fingerprints. The subsequent permutation test further revealed that the observed cosine similarity between the two CR fingerprints was greater than the calculated test criterion at the 5% significance level in the right tail. ‘n.s.’ indicates that no significant difference was found after a paired samples *t*-test in subsequent single connection analyses.

Two ventral striatum-like subregions, namely the Acb-like and NUDAPs-like divisions, were characterized using the CR fingerprint in both species (Figure 4). When comparing the Acb-like and NUDAPs-like divisions within a species, the permutation tests indicated that the observed cosine similarity between the two fingerprints of the two subregions was greater than the calculated test criterion at the 5% significance level in the right tail, i.e., the two fingerprints are ‘far’ from each other. Therefore, we rejected the null hypothesis and indicated that the two ventral striatum-like subregions have distinct anatomical connectivity profiles. More specifically, the Acb-like division, when compared to the NUDAPs-like division, was found to have significantly stronger anatomical connectivities with all the above-mentioned targets in the single connection analyses, except for areas 13, 14, INS, TP, and Pu. In contrast, the latter two targets (TP and Pu) presented stronger connectivities with the NUDAPs-like division. An absence of significant interspecies connectivity differences was found for the targets of areas 13, 14, and INS.

**FIGURE 5:**
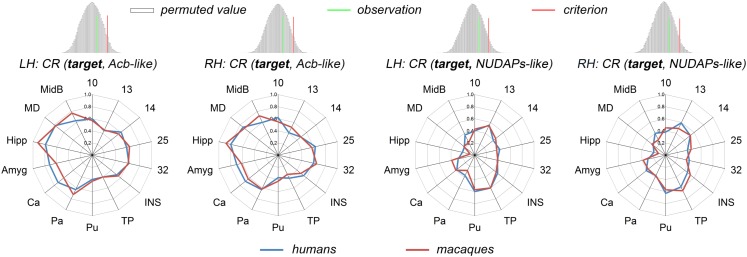
Comparison between humans and macaques using the CR fingerprints of ventral striatal components. The CR fingerprints of the human and macaque ventral striatum-like subregions, the Acb-like and NUDAPs-like divisions, are compared and displayed in the left and right two panels, respectively. They all present convergent fingerprints given that the observed cosine similarity was lesser than the significance criterion in the right tail shown in the permutation tests.

The corresponding CR fingerprints of the Acb-like and NUDAPs-like divisions between the two species were extracted and compared to detect their cosine similarity. The permutation tests indicated that the observed cosine similarity between the two ratio fingerprints was not a rare value in the permutation distribution, as can be seen in the histogram. The observed cosine similarity was lesser than the calculated test criterion at the 5% significance level in the right tail (Figure 5). Thus, we accepted the null hypothesis and indicated that the two CR fingerprints are ‘close’ to each other. This result suggested that the gross connectivity trend of these targets to the two ventral striatum-like subregions is conserved between the two species. In other words, both connectional strengthening and weakening in the Acb-like and NUDAPs-like divisions occurred uniformly in humans and macaques.

## Discussion

Both the human striatum and macaque striatum were recursively parcellated based on tractography. The ventral striatum-like, Acb-like, and NUDAPs-like connection units were defined in both species and treated as seeds for cross-species comparisons. A set of areas meeting specific criteria was extracted as targets to construct connectivity fingerprints, which were used as the comparative characteristics to characterize and compare the seeds. Our results revealed that the RCS fingerprint of the ventral striatum-like division in humans differed considerably from that of macaques. However, the general connectivity trend of these targets to the ventral striatum-like subregions, the Acb-like and NUDAPs-like divisions, was conserved, based on their convergent CR fingerprints.

### Cross-Species Comparative Basis

The gross correspondence between connectivity architecture and microstructural features (e.g., cyto- and myelo-architecture) has yet to be clarified; in fact, both similar (Barbas and Rempel-Clower, 1997; van den Heuvel et al., 2015) and discrepant (Jongen-Rêlo et al., 1994; Moerel et al., 2014) corresponding results exist in previous studies. For example, Jongen-Rêlo et al. (1994) found considerable differences in the delineation of the boundaries of the ventral striatal components (e.g., the Acb shell/core borders) in substance P immunoreactivity and acetylcholinesterase patterns and in the calbindin-d28k immunoreactivity pattern. Therefore, the resulting connection unit in the current study involved voxels that showed similar whole-brain anatomical connectivity architecture, which is considered more suitable for connection analyses than microstructurally-defined regions. Furthermore, since function may depend considerably more on connectivity architecture than on microstructural features (Knosche and Tittgemeyer, 2011; Cloutman and Lambon Ralph, 2012; Glickfeld et al., 2013), the delineation of the connection units of the ventral striatal components is crucially important for reliably dealing with the ever-increasing number of connection analyses conducted on these regions. In addition, the connection units generated in both species provide an excellent basis for viable interspecies comparisons using the connectivity characteristics.

### Comparison of the Ventral Striatal Components

As the connectivity-based ventral striatum-like division (Tziortzi et al., 2013; Janssen et al., 2015; Fan et al., 2016) covers a much wider area than the ventral striatum proper (Haber and McFarland, 1999; Heimer et al., 1999), it may be more appropriate to refer to it as the ‘limbic-related striatum,’ as in previous rat studies (Kelley and Domesick, 1982; Kelley et al., 1982; Kita and Kitai, 1990). Both high-quality human HCP MRI data and high-resolution *ex vivo* macaque MRI data enabled the accurate identification of both the ventral striatum-like and the Acb-like connection units in both species. The lateral ventral striatum (i.e., the ventral one-third Pu) was excluded from the Acb-like division given its large localization error with the histological Acb, and was called the NUDAPs-like division only because the histologically-defined patch-like NUDAPs defined in histology is mostly distributed in this region (Voorn et al., 1996; Daunais et al., 2001). Furthermore, because the cross-species comparison of the Acb shell/core architecture was detailed in our previous study (in preparation), further identification of the Acb subregions was not done; only the corresponding ventral striatum-like, Acb-like, and NUDAPs-like divisions between the two species were compared.

The most first finding was the similar topographical distribution of these connectivity-based ventral striatal structures between the two primate brains. This finding supplemented previous research that showed similarities in their cellular and molecular composition and distribution, axonal projections, and functions across species (Izawa et al., 2003; Basar et al., 2010; Daniel and Pollmann, 2014; Salgado and Kaplitt, 2015). In subsequent connection analyses, dissimilar interspecies RCS fingerprints, i.e., the macroscopic white matter tracts layout, of the ventral striatum-like division were also found. This result aligns with disproportionate volumetric differences in the regions associated with the ventral striatum, including both the disproportionate growth of the human prefrontal cortex (Carlén, 2017; Smaers et al., 2017), prefrontal white matter (Schoenemann et al., 2005), Hipp and Amyg (Barger et al., 2014) during primate evolution, and the significantly smaller human striatum (which may influence the ventral striatum) than those predicted based on primate scaling trends (Barger et al., 2014). More importantly, this interspecies connectivity difference may suggest a functional modification of the ventral striatum, which was inferred and is summarized as follows.

### Possible Interspecies Functional Modifications

The infralimbic area is involved in representing primary rewards (e.g., food) and the decision-making process via its projection to the ventral striatum (Kringelbach and Rolls, 2004; McNamee et al., 2013; Sescousse et al., 2013). A significant interspecies difference in connectivity between area 25 and the ventral striatum-like division was not detected, suggesting a similar processing ability of humans and macaques in response to primary rewards. In contrast, the human ventral striatum-like division has prominent connectivity with areas 10 and 14, indicating a better processing ability for abstract rewards and an increased abstractness in humans compared to macaques (Kringelbach and Rolls, 2004; McNamee et al., 2013; Sescousse et al., 2013). The projection from the prelimbic area to the ventral striatal patch pathway was identified as being required for decision-making in conflict situations (Friedman et al., 2015). The significantly stronger connectivity between the ventral striatum-like division and area 32 seen in the macaques, when compared to humans, may suggest a greater ability of macaques to adjust or switch reward-related actions in conflict situations. The Amyg projection to the ventral striatum appears to be involved in both the encoding of stimuli values and the prediction of their appetitive or aversive consequences, thus enabling the adjustment of their motivational level (Russchen et al., 1985; Cardinal and Everitt, 2004). Furthermore, the significant difference in this connection between the two species may imply a larger number of stimuli or/and an overcomplication of mood-related encoding of such stimuli when processed in humans. The thalamus is the hub of the cortico-striato-thalamo-cortical spiral-like loop (Loonen and Ivanova, 2016), and the connection between the mediodorsal nucleus and the Acb core was found to be necessary for the expression of aversive withdrawal symptoms (Zhu et al., 2016). The stronger connectivity between the ventral striatum-like area and the MD seen in humans compared with macaques may suggest a good ability of humans to predict and avoid aversive consequences.

Although dissimilar RCS fingerprints of the ventral striatum-like division and some significantly different single connections were revealed between humans and macaques, conservative connectivity trends of these targets to the Acb-like and NUDAPs-like divisions were present. The synchronous changed (strengthen or weaken) connectivity observed in the two subregions in both types of primate brains indicated that the labor division and the cooperation of the two ventral striatum subregions may have not been noticeably altered across species.

## Conclusion

The same tractography-based recursive parcellations of the striatum were performed in both humans and macaques to delineate their ventral striatum-like, Acb-like, and NUDAPs-like connection units. A set of targets was chosen to enable the construction of fingerprints to characterize and compare such components across species. A dissimilar RCS fingerprint of the ventral striatum-like division was revealed between the species. Furthermore, this connectivity difference was localized to specific targets and such single connectivity differences were used to infer possible interspecies functional modifications. Finally, our results revealed distinct connectivity fingerprints of the Acb-like and NUDAPs-like divisions and interspecies convergent CR fingerprints of this target group to the two ventral striatum-like subregions.

## Ethics Statement

Eight adult rhesus macaque brain specimens were used in this study. These monkeys were judged by the veterinarian as appropriate subjects for euthanasia due to the presence of serious physical diseases. All the brain specimens were obtained at necropsy immediately following euthanasia due to reasons not related to the study and acquired following protocols approved by the National Animal Research Authority of China. All experimental procedures were performed in strict accordance with the recommendations in the National Institutes of Health Guide for the Care and Use of Laboratory Animals. All of the animals were handled according to the protocol (#IA-2016-05) approved by the Animal Care and Use Committee of the Institute of Automation, Chinese Academy of Sciences.

## Author Contributions

TJ and HL conceived and designed the experiments. XX, LF, CC, RY, HD, and DZ carried out the research and analyzed the data. XX, LF, HL, and TJ wrote the manuscript. All authors reviewed and approved the manuscript for submission.

## Conflict of Interest Statement

The authors declare that the research was conducted in the absence of any commercial or financial relationships that could be construed as a potential conflict of interest.

## References

[B1] Avants B. B. Epstein C. L. Grossman M. Gee J. C. (2008). Symmetric diffeomorphic image registration with cross-correlation: evaluating automated labeling of elderly and neurodegenerative brain. *Med. Image Anal.* 12 26–41. 10.1016/j.media.2007.06.004 17659998 PMC2276735

[B2] Barbas H. Rempel-Clower N. (1997). Cortical structure predicts the pattern of corticocortical connections. *Cereb. Cortex* 7 635–646. 10.1093/cercor/7.7.635 9373019

[B3] Barger N. Hanson K. L. Teffer K. Schenker-Ahmed N. M. Semendeferi K. (2014). Evidence for evolutionary specialization in human limbic structures. *Front. Hum. Neurosci.* 8:e277. 10.3389/fnhum.2014.00277 24904348 PMC4033018

[B4] Basar K. Sesia T. Groenewegen H. Steinbusch H. W. M. Visser-Vandewalle V. Temel Y. (2010). Nucleus accumbens and impulsivity. *Prog. Neurobiol.* 92 533–557. 10.1016/j.pneurobio.2010.08.007 20831892

[B5] Beckmann M. Johansen-Berg H. Rushworth M. F. (2009). Connectivity-based parcellation of human cingulate cortex and its relation to functional specialization. *J. Neurosci.* 29 1175–1190. 10.1523/JNEUROSCI.3328-08.2009 19176826 PMC6665147

[B6] Behrens T. E. Johansen-Berg H. Woolrich M. W. Smith S. M. Wheeler-Kingshott C. A. Boulby P. A. (2003). Non-invasive mapping of connections between human thalamus and cortex using diffusion imaging. *Nat. Neurosci.* 6 750–757. 10.1038/nn1075 12808459

[B7] Blomstedt P. Sjöberg R. L. Hansson M. Bodlund O. Hariz M. I. (2013). Deep brain stimulation in the treatment of obsessive-compulsive disorder. *World Neurosurg.* 80 245–253. 10.1016/j.wneu.2012.10.006 23044000

[B8] Brauer K. Hausser M. Hartig W. Arendt T. (2000). The core-shell dichotomy of nucleus accumbens in the rhesus monkey as revealed by double-immunofluorescence and morphology of cholinergic interneurons. *Brain. Res.* 858 151–162. 10.1016/S0006-8993(00)01938-7 10700608

[B9] Calabrese E. Badea A. Coe C. L. Lubach G. R. Shi Y. Styner M. A. (2015). A diffusion tensor MRI atlas of the postmortem rhesus macaque brain. *Neuroimage* 117 408–416. 10.1016/j.neuroimage.2015.05.072 26037056 PMC4512905

[B10] Calipari E. S. Huggins K. N. Mathews T. A. Jones S. R. (2012). Conserved dorsal-ventral gradient of dopamine release and uptake rate in mice, rats and rhesus macaques. *Neurochem. Int.* 61 986–991. 10.1016/j.neuint.2012.07.008 22819794 PMC3593229

[B11] Cardinal R. N. Everitt B. J. (2004). Neural and psychological mechanisms underlying appetitive learning: links to drug addiction. *Curr. Opin. Neurobiol.* 14 156–162. 10.1016/j.conb.2004.03.004 15082319

[B12] Carlén M. (2017). What constitutes the prefrontal cortex? *Science* 358 478–482. 10.1126/science.aan8868 29074767

[B13] Caspers S. Eickhoff S. B. Geyer S. Scheperjans F. Mohlberg H. Zilles K. (2008). The human inferior parietal lobule in stereotaxic space. *Brain. Struct. Funct.* 212 481–495. 10.1007/s00429-008-0195-z 18651173

[B14] Cloutman L. L. Lambon Ralph M. A. (2012). Connectivity-based structural and functional parcellation of the human cortex using diffusion imaging and tractography. *Front. Neuroanat.* 6:e34 10.3389/fnana.2012.00034 PMC3429885 22952459

[B15] Daniel R. Pollmann S. (2014). A universal role of the ventral striatum in reward-based learning: evidence from human studies. *Neurobiol. Learn. Mem.* 114 90–100. 10.1016/j.nlm.2014.05.002 24825620 PMC4143465

[B16] Daunais J. B. Letchworth S. R. Sim-Selley L. J. Smith H. R. Childers S. R. Porrino L. J. (2001). Functional and anatomical localization of mu opioid receptors in the striatum, amygdala, and extended amygdala of the nonhuman primate. *J. Comp. Neurol.* 433 471–485. 10.1002/cne.1154 11304712

[B17] Desikan R. S. Segonne F. Fischl B. Quinn B. T. Dickerson B. C. Blacker D. (2006). An automated labeling system for subdividing the human cerebral cortex on MRI scans into gyral based regions of interest. *Neuroimage* 31 968–980. 10.1016/j.neuroimage.2006.01.021 16530430

[B18] Eickhoff S. B. Heim S. Zilles K. Amunts K. (2006). Testing anatomically specified hypotheses in functional imaging using cytoarchitectonic maps. *Neuroimage* 32 570–582. 10.1016/j.neuroimage.2006.04.204 16781166

[B19] Fan L. Li H. Zhuo J. Zhang Y. Wang J. Chen L. (2016). The human brainnetome atlas: a new brain atlas based on connectional architecture. *Cereb. Cortex* 26 3508–3526. 10.1093/cercor/bhw157 27230218 PMC4961028

[B20] Floresco S. B. (2015). The nucleus accumbens: an interface between cognition, emotion, and action. *Annu. Rev. Psychol.* 66 25–52. 10.1146/annurev-psych-010213-115159 25251489

[B21] Frey S. Pandya D. N. Chakravarty M. M. Bailey L. Petrides M. Collins D. L. (2011). An MRI based average macaque monkey stereotaxic atlas and space (MNI monkey space). *Neuroimage* 55 1435–1442. 10.1016/j.neuroimage.2011.01.040 21256229

[B22] Friedman A. Homma D. Gibb L. G. Amemori K. Rubin S. J. Hood A. S. (2015). A corticostriatal path targeting striosomes controls decision-making under conflict. *Cell* 161 1320–1333. 10.1016/j.cell.2015.04.049 26027737 PMC4477966

[B23] Gelfand Y. Kaplitt M. G. (2013). Gene therapy for psychiatric disorders. *World Neurosurg.* 80 11–18. 10.1016/j.wneu.2012.12.028 23268195

[B24] Glickfeld L. L. Andermann M. L. Bonin V. Reid R. C. (2013). Cortico-cortical projections in mouse visual cortex are functionally target specific. *Nat. Neurosci.* 16 219–226. 10.1038/nn.3300 23292681 PMC3808876

[B25] Greve D. N. Fischl B. (2009). Accurate and robust brain image alignment using boundary-based registration. *Neuroimage* 48 63–72. 10.1016/j.neuroimage.2009.06.060 19573611 PMC2733527

[B26] Haber S. N. McFarland N. R. (1999). The concept of the ventral striatum in nonhuman primates. *Ann. N. Y. Acad. Sci.* 877 33–48. 10.1111/j.1749-6632.1999.tb09259.x 10415641

[B27] Hecht E. E. Gutman D. A. Bradley B. A. Preuss T. M. Stout D. (2015). Virtual dissection and comparative connectivity of the superior longitudinal fasciculus in chimpanzees and humans. *Neuroimage* 108 124–137. 10.1016/j.neuroimage.2014.12.039 25534109 PMC4324003

[B28] Heilbronner S. R. Rodriguez-Romaguera J. Quirk G. J. Groenewegen H. J. Haber S. N. (2016). Circuit-based corticostriatal homologies between rat and primate. *Biol. Psychiatry* 80 509–521. 10.1016/j.biopsych.2016.05.012 27450032 PMC5438202

[B29] Heimer L. De Olmos J. S. Alheid G. F. Person J. Sakamoto N. Shinoda K. (1999). “The human basal forebrain. Part II,” in *Handbook of Chemical Neuroanatomy*, eds Bloom F. E. Bjorkland A. Hokfelt T. (Amsterdam: Elsevier), 57–226. 10.1016/s0924-8196(99)80024-4

[B30] Izawa E. Zachar G. Yanagihara S. Matsushima T. (2003). Localized lesion of caudal part of lobus parolfactorius caused impulsive choice in the domestic chick: evolutionarily conserved function of ventral striatum. *J. Neurosci.* 23 1894–1902. 10.1523/JNEUROSCI.23-05-01894.2003 12629194 PMC6741993

[B31] Janssen R. J. Jylanki P. Kessels R. P. C. van Gerven M. A. J. (2015). Probabilistic model-based functional parcellation reveals a robust, fine-grained subdivision of the striatum. *Neuroimage* 119 398–405. 10.1016/j.neuroimage.2015.06.084 26163800

[B32] Johansen-Berg H. Behrens T. E. Robson M. D. Drobnjak I. Rushworth M. F. Brady J. M. (2004). Changes in connectivity profiles define functionally distinct regions in human medial frontal cortex. *Proc. Natl. Acad. Sci. U.S.A.* 101 13335–13340. 10.1073/pnas.0403743101 15340158 PMC516567

[B33] Jongen-Rêlo A. L. Voorn P. Groenewegen H. J. (1994). Immunohistochemical characterization of the shell and core territories of the nucleus accumbens in the rat. *Eur. J. Neurosci.* 6 1255–1264. 10.1111/j.1460-9568.1994.tb00315.x 7526940

[B34] Kelley A. E. Domesick V. B. (1982). The distribution of the projection from the hippocampal formation to the nucleus accumbens in the rat: an anterograde- and retrograde-horseradish peroxidase study. *Neuroscience* 7 2321–2335. 10.1016/0306-4522(82)90198-1 6817161

[B35] Kelley A. E. Domesick V. B. Nauta W. J. (1982). The amygdalostriatal projection in the rat - an anatomical study by anterograde and retrograde tracing methods. *Neuroscience* 7 615–630. 10.1016/0306-4522(82)90067-7 7070669

[B36] Kita H. Kitai S. T. (1990). Amygdaloid projections to the frontal cortex and the striatum in the rat. *J. Comp. Neurol.* 298 40–49. 10.1002/cne.902980104 1698828

[B37] Knosche T. R. Tittgemeyer M. (2011). The role of long-range connectivity for the characterization of the functional-anatomical organization of the cortex. *Front. Syst. Neurosci.* 5:e58. 10.3389/fnsys.2011.00058 21779237 PMC3133730

[B38] Kringelbach M. L. Rolls E. T. (2004). The functional neuroanatomy of the human orbitofrontal cortex: evidence from neuroimaging and neuropsychology. *Prog. Neurobiol.* 72 341–372. 10.1016/j.pneurobio.2004.03.006 15157726

[B39] Li H. Fan L. Zhuo J. Wang J. Zhang Y. Yang Z. (2017). ATPP: a pipeline for automatic tractography-based brain parcellation. *Front. Neuroinform.* 11:e35. 10.3389/fninf.2017.00035 28611620 PMC5447055

[B40] Liu H. Qin W. Li W. Fan L. Wang J. Jiang T. (2013). Connectivity-based parcellation of the human frontal pole with diffusion tensor imaging. *J. Neurosci.* 33 6782–6790. 10.1523/JNEUROSCI.4882-12.2013 23595737 PMC6618893

[B41] Loonen A. J. Ivanova S. A. (2016). Circuits regulating pleasure and happiness in major depression. *Med. Hypotheses.* 87 14–21. 10.1016/j.mehy.2015.12.013 26826634

[B42] Mars R. B. Foxley S. Verhagen L. Jbabdi S. Sallet J. Noonan M. P. (2016a). The extreme capsule fiber complex in humans and macaque monkeys: a comparative diffusion MRI tractography study. *Brain Struct. Funct.* 221 4059–4071. 10.1007/s00429-015-1146-0 26627483 PMC5065901

[B43] Mars R. B. Verhagen L. Gladwin T. E. Neubert F.-X. Sallet J. Rushworth M. F. (2016b). Comparing brains by matching connectivity profiles. *Neurosci. Biobehav. Rev.* 60 90–97. 10.1016/j.neubiorev.2015.10.008 26627865 PMC6485474

[B44] Mars R. B. Passingham R. E. Jbabdi S. (2018a). Connectivity fingerprints: from areal descriptions to abstract spaces. *Trends Cogn. Sci.* 22 1026–1037. 10.1016/j.tics.2018.08.009 30241910 PMC6198109

[B45] Mars R. B. Sotiropoulos S. N. Passingham R. E. Sallet J. Verhagen L. Khrapitchev A. A. (2018b). Whole brain comparative anatomy using connectivity blueprints. *eLife* 7:e35237. 10.7554/eLife.35237 29749930 PMC5984034

[B46] Mars R. B. Sallet J. Schüffelgen U. Jbabdi S. Toni I. Rushworth M. F. (2012). Connectivity-based subdivisions of the human right “temporoparietal junction area”: evidence for different areas participating in different cortical networks. *Cereb. Cortex* 22 1894–1903. 10.1093/cercor/bhr268 21955921

[B47] McNamee D. Rangel A. O’Doherty J. P. (2013). Category-dependent and category-independent goal-value codes in human ventromedial prefrontal cortex. *Nat. Neurosci.* 16 479–485. 10.1038/nn.3337 23416449 PMC3665508

[B48] Moerel M. De Martino F. Formisano E. (2014). An anatomical and functional topography of human auditory cortical areas. *Front. Neurosci.* 8:225. 10.3389/fnins.2014.00225 25120426 PMC4114190

[B49] Murty V. P. Shermohammed M. Smith D. V. Carter R. M. Huettel S. A. Adcock R. A. (2014). Resting state networks distinguish human ventral tegmental area from substantia nigra. *Neuroimage* 100 580–589. 10.1016/j.neuroimage.2014.06.047 24979343 PMC4370842

[B50] Neubert F. Mars R. B. Sallet J. Rushworth M. F. S. (2015). Connectivity reveals relationship of brain areas for reward-guided learning and decision making in human and monkey frontal cortex. *Proc. Natl. Acad. Sci. U.S.A.* 112 2695–2704. 10.1073/pnas.1410767112 25947150 PMC4443352

[B51] Passingham R. E. Stephan K. E. Kotter R. (2002). The anatomical basis of functional localization in the cortex. *Nat. Rev. Neurosci.* 3 606–616. 10.1038/nrn893 12154362

[B52] Paxinos G. Huang X.-F. Toga A. W. (2009). *The Rhesus Monkey Brain in Stereotaxic Coordinates*, 2nd Edn San Diego, CA: Academic Press.

[B53] Petrides M. Tomaiuolo F. Yeterian E. H. Pandya D. N. (2012). The prefrontal cortex: comparative architectonic organization in the human and the macaque monkey brains. *Cortex* 48 46–57. 10.1016/j.cortex.2011.07.002 21872854

[B54] Rilling J. K. Glasser M. F. Jbabdi S. Andersson J. Preuss T. M. (2011). Continuity, divergence, and the evolution of brain language pathways. *Front. Evol. Neurosci.* 3:11. 10.3389/fnevo.2011.00011 22319495 PMC3249609

[B55] Rilling J. K. Glasser M. F. Preuss T. M. Ma X. Zhao T. Hu X. (2008). The evolution of the arcuate fasciculus revealed with comparative DTI. *Nat. Neurosci.* 11 426–428. 10.1038/nn2072 18344993

[B56] Russchen F. T. Bakst I. Amaral D. G. Price J. (1985). The amygdalostriatal projections in the monkey. an anterograde tracing study. *Brain Res.* 329 241–257. 10.1016/0006-8993(85)90530-X 3978445

[B57] Salgado S. Kaplitt M. G. (2015). The nucleus accumbens: a comprehensive review. *Stereotact. Funct. Neurosurg.* 93 75–93. 10.1159/000368279 25720819

[B58] Schoenemann P. T. Sheehan M. J. Glotzer L. D. (2005). Prefrontal white matter volume is disproportionately larger in humans than in other primates. *Nat. Neurosci.* 8 242–252. 10.1038/nn1394 15665874

[B59] Sescousse G. Caldú X. Segura B. Dreher J.-C. (2013). Processing of primary and secondary rewards: a quantitative meta-analysis and review of human functional neuroimaging studies. *Neurosci. Biobehav. Rev.* 37 681–696. 10.1016/j.neubiorev.2013.02.002 23415703

[B60] Smaers J. B. Gómez-Robles A. Parks A. N. Sherwood C. C. (2017). Exceptional evolutionary expansion of prefrontal cortex in great apes and humans. *Curr. Biol.* 27 714–720. 10.1016/j.cub.2017.01.020 28162899

[B61] Somel M. Liu X. Tang L. Yan Z. Hu H. Guo S. (2011). MicroRNA-driven developmental remodeling in the brain distinguishes humans from other primates. *PLoS. Biol.* 9:e1001214. 10.1371/journal.pbio.1001214 22162950 PMC3232219

[B62] Sotiropoulos S. N. Jbabdi S. Xu J. Andersson J. L. Moeller S. Auerbach E. J. (2013). Advances in diffusion MRI acquisition and processing in the human connectome project. *Neuroimage* 80 125–143. 10.1016/j.neuroimage.2013.05.057 23702418 PMC3720790

[B63] Sousa A. M. M. Zhu Y. Raghanti M. A. Kitchen R. R. Onorati M. Tebbenkamp A. T. N. (2017). Molecular and cellular reorganization of neural circuits in the human lineage. *Science* 358 1027–1032. 10.1126/science.aan3456 29170230 PMC5776074

[B64] Sturm V. Lenartz D. Koulousakis A. Treuer H. Herholz K. Klein J. C. (2003). The nucleus accumbens: a target for deep brain stimulation in obsessive–compulsive- and anxietydisorders. *J. Chem. Neuroanat.* 26 293–299. 10.1016/j.jchemneu.2003.09.003 14729131

[B65] Thiebaut de Schotten M. Croxson P. L. Mars R. B. (2018). Large-scale comparative neuroimaging: where are we and what do we need? *Cortex* [Epub ahead of print]. 30661736 10.1016/j.cortex.2018.11.028 PMC6699599

[B66] Tomassini V. Jbabdi S. Klein J. C. Behrens T. E. J. Pozzilli C. Matthews P. M. (2007). Diffusion-weighted imaging tractography-based parcellation of the human lateral premotor cortex identifies dorsal and ventral subregions with anatomical and functional specializations. *J. Neurosci.* 27 10259–10269. 10.1523/JNEUROSCI.2144-07.2007 17881532 PMC6672665

[B67] Tziortzi A. C. Haber S. N. Searle G. E. Tsoumpas C. Long C. J. Shotbolt P. (2013). Connectivity-based functional analysis of dopamine release in the striatum using diffusion-weighted MRI and positron emission tomography. *Cereb. Cortex* 24 1165–1177. 10.1093/cercor/bhs397 23283687 PMC3977617

[B68] Ugurbil K. Xu J. Auerbach E. J. Moeller S. Vu A. T. Duarte-Carvajalino J. M. (2013). Pushing spatial and temporal resolution for functional and diffusion MRI in the human connectome project. *Neuroimage* 80 80–104. 10.1016/j.neuroimage.2013.05.012 23702417 PMC3740184

[B69] van den Heuvel M. P. Scholtens L. H. Feldman B. Hilgetag C. C. de Reus M. A. (2015). Bridging cytoarchitectonics and connectomics in human cerebral cortex. *J. Neurosci.* 35 13943–13948. 10.1523/JNEUROSCI.2630-15.2015 26468195 PMC6608182

[B70] Van Essen D. C. Smith S. M. Barch D. M. Behrens T. E. Yacoub E. Ugurbil K. (2013). The WU-minn human connectome project: an overview. *Neuroimage* 80 62–79. 10.1016/j.neuroimage.2013.05.041 23684880 PMC3724347

[B71] Volkow N. D. Morales M. (2015). The brain on drugs: from reward to addiction. *Cell* 162 712–725. 10.1016/j.cell.2015.07.046 26276628

[B72] Voorn P. Brady L. S. Berendse H. W. Richfield E. K. (1996). Densitometrical analysis of opioid receptor ligand binding in the human striatum – I. Distribution of u opioid receptor defines shell and core of the ventral striatum. *Neuroscience* 75 777–792. 10.1016/0306-4522(96)00271-0 8951872

[B73] Xia X. Fan L. Cheng C. Eickhoff S. B. Chen J. Li H. (2017). Multimodal connectivity-based parcellation reveals a shell-core dichotomy of the human nucleus accumbens. *Hum. Brain. Mapp* 38 3878–3898. 10.1002/hbm.23636 28548226 PMC5685173

[B74] Zhang D. Guo L. Zhu D. Li K. Li L. Chen H. (2013). Diffusion tensor imaging reveals evolution of primate brain architectures. *Brain. Struct. Funct.* 218 1429–1450. 10.1007/s00429-012-0468-4 23135357 PMC3663907

[B75] Zhu Y. Wienecke C. F. Nachtrab G. Chen X. (2016). A thalamic input to the nucleus accumbens mediates opiate dependence. *Nature* 530 219–222. 10.1038/nature16954 26840481 PMC4814115

[B76] Zhuo J. Fan L. Liu Y. Zhang Y. Yu C. Jiang T. (2016). Connectivity profiles reveal a transition subarea in the parahippocampal region that integrates the anterior temporal-posterior medial systems. *J. Neurosci.* 36 2782–2795. 10.1523/JNEUROSCI.1975-15.2016 26937015 PMC6604873

