# Supplementary material for: Interspecies Differences in the Connectivity of Ventral Striatal Components Between Humans and Macaques
Source: Front Neurosci. 2019 Jun 14;13:623. doi: 10.3389/fnins.2019.00623 (PMC6587664; doi:10.3389/fnins.2019.00623)
Supplement: Supplementary file 1 [file Data_Sheet_1.docx]

Supplementary Material

# Anatomical Boundaries of the Acb

The ventral striatum is often synonymous with the Acb in anatomy (Heimer et al. 1999). The boundaries of the anatomical Acb, suggested by more modern anatomical analyses, could be described as follows (Salgado and Kaplitt, 2015): (1) posterior limit: the posterior border of the anterior commissure (Heimer, 2000; Neto et al., 2008); (2) anterior limit: where the rostral limit of the internal capsule starts separating the caudate from the putamen (Neto et al., 2008); (3) medial limit: the sagittal plane passing by the inferior border of the lateral ventricle; (4) lateral limit: a line extending downwards and laterally to the rostral edge of the internal capsule; (5) dorsal limit: the horizontal plane passing under the caudate nucleus head from the inferior border of the lateral ventricle to the inferior limit of the internal capsule, and (6) ventral limit: the external capsule (lateral side) and Broca’s diagonal band (medial side) anteriorly, the anterior hypothalamic nucleus posteriorly (Neto et al., 2008).

# Preparation of the Specimens and MRI Data Acquisition

High-resolution *ex vivo* rhesus macaque MRI dataset consisted of 8 adult rhesus macaque brain specimens (Table S1). These monkeys were obtained from Kunming Institute of Zoology, Chinese Academy of Sciences, and were judged by the veterinarian as appropriate subjects for euthanasia due to serious physical diseases. The macaques were administered an overdose of pentobarbital (100 mg/kg). After verifying the status of deep anesthesia, they were first transcardially perfused with phosphate-buffered saline (PBS) solution containing 1% heparin (PH7.4), followed by a pre-cooled PBS solution containing 4% paraformaldehyde. 5 minutes after beginning the perfusion, the speed was lowered to 1 ml/min, which was maintained for 2 hours. The heads were then removed from the bodies and stored in PBS solution containing 4% paraformaldehyde. The skull was carefully removed to expose the whole brain and was transferred to an MRI compatible holder, bundled with medical gauze, and immersed in Fomblin (Solvay, Brussels, Belgium) to prevent dehydration and susceptibility to artifacts. No apparent structural anomaly was found in any of the brains used in the present study. MRI data were performed on a 9.4T horizontal animal MRI system (Bruker Biospec 94/30 USR) with Paravision 6.0.1, the gradients are equipped with slew rate of 1170 mT/m/ms and maximum strength 300 mT/m, radiofrequency transmission and reception were achieved with a 154 mm inner-diameter quadrature radiofrequency coil. T2w images were acquired using 2D Turbo RARE sequence with TR/TE = 8464/30.9 ms, flip angle = 90°, matrix = 280×220, FOV = 84×66 mm, slice thickness = 0.6mm, number of excitations = 4. DTI images were acquired using a 2D diffusion weighted spin echo pulse sequence, TR/TE = 9800/21.8 ms, bandwidth = 304 kHz, FOV = 94×66 mm, matrix = 140×110, and slice thickness = 0.6 mm. 60 diffusion directions with b = 1000 s/mm^2^ (Δ/δ = 10.9/4.5 ms, maximum gradient amplitude = 276 mT/m) and 4 non-diffusion gradients (b = 0 s/mm^2^) acquisition, and time cost was approximately 115 hours per specimen. The data quality of these diffusion images were analyzed in Section 3.

**Table S1.** Descriptive information for the 8 rhesus macaque brain specimens

| **Perfusion date (year-month-day)** | **No.** | **Sex** | **Age (years)** | **Body weight (kg)** |
| --- | --- | --- | --- | --- |
| 2016-05-09 | 93310 | female | 23 | 4.24 |
|  | 08046 | female | 8 | 4.58 |
|  | 12027 | male | 4 | 4.06 |
|  | 12411 | male | 4 | 3.89 |
| 2016-05-10 | 01006 | female | 15 | 4.57 |
|  | 04084 | female | 12 | 5.23 |
|  | 10427 | female | 6 | 4.69 |
|  | 11402 | female | 5 | 3.90 |

* Note: Their weights are significantly lower than the normal rhesus macaque due to physical diseases.

# MRI Data Quality Checking

The visual inspection was first performed for these MRI data to ensure there were no artefacts and geometric distortions caused by the static magnetic field inhomogeneity and the eddy current, etc.

For the *ex vivo* macaque diffusion MRI data, the *b*-value was not set to the 4000 s/mm^2^ recommended in previous studies (D’Arceuil et al., 2007; Dyrby et al., 2011) but at the relatively low value of 1000 s/mm^2^ like some other studies (Table S2). For example, Calabrese et al. (2015) reconstructed 42 major white matter tracks and even several smaller pathways (e.g., the cranial nerves, the fasciculus retroflexus, and the stria medullaris) using diffusion images with the relatively low *b*-value of 1500 s/mm^2^. In this study, a preliminary test of the data quality was performed for these diffusion images using a typical signal-to-noise ratio (SNR). Further testing was performed by comparing the parcellation results generated using different *b*-values (1000, 2000, 3000, and 4000 s/mm^2^) for the diffusion images.

**Table S2.** Previous studies using relatively low *b*-value for tractographic reconstruction

| **Authors** | **Scanner** | **Subjects** | **DTI Sequence** | **TR/TE (ms)** | **# Dir.** | **Max *b*-value (s/mm^2^) & other parameters** |
| --- | --- | --- | --- | --- | --- | --- |
| Calabrese et al., 2016 | Agilent 7T field strength 650 mT/m gradient 65mm QUA. Coil | 10 rhesus monkey brain specimens | 3D diffusion weighted spin echo | 100/21.5 | 12 | **1500** Δ = 14 ms δ = 4 ms amplitude = 50 G/cm |
| Rane et al., 2010 | Siemens TRIO 3T | 4 rhesus monkey brain specimens | STEAM EPI; STEAM EPI; double spin-echo | 1500/80; 1500/80; 1500/104 | 30; 30; 30 | **1700**, δ = 21 ms, diffusion time = 48 ms; **1700**, δ = 21 ms, diffusion time = 192 ms; **1700**, δ = 21 ms, effective diffusion time = 45 ms |
| Budde et al., 2011 | Bruker Biospec 7T field strength;  microimaging gradient insert and a 20-mm probe. | 5 (8-12 week-old; female) Wistar rats brain specimens | A multiple-echo pulsed gradient spin echo sequence | 4000/20 | 30 | **1200** Δ = 10 ms δ = 4 ms |
| Feng et al., 2017 | A Bruker 4.7 T scanner | 10 (age: 5.3 ± 2.8 years; body; weight = 5.67 ± 2.34 kg; 6 male) macaques | 3D multiple spin echo diffusion tensor sequence | 700/32.5 | 8 | **1000** |

Because the weak diffusion-weighted signal produced by a low *b*-value magnetic field can be close to the background noise level, the resulting insufficient SNR can present a critical problem for the subsequent tractographic reconstruction and eventual parcellation. The SNRs of the macaque diffusion images were calculated and checked using a single image-based two-region method (Dietrich et al., 2007; Griffanti et al., 2012). Specifically, for each macaque, two separate tissues, the gray matter (GM) and white matter (WM), were extracted in subject-native diffusion space to estimate the signals; a region positioned in the background was used to evaluate the noise. The SNRs were then calculated for these diffusion images (Figure S1). As a rule of thumb, the SNR of the diffusion MRI acquisition image should be at least 20 to derive relatively unbiased measures of parameters, such as the fractional anisotropy (Mukherjee et al., 2008). All the diffusion images in this study met this requirement and thus had an appropriate SNR for the subsequent tractographic reconstruction.

**
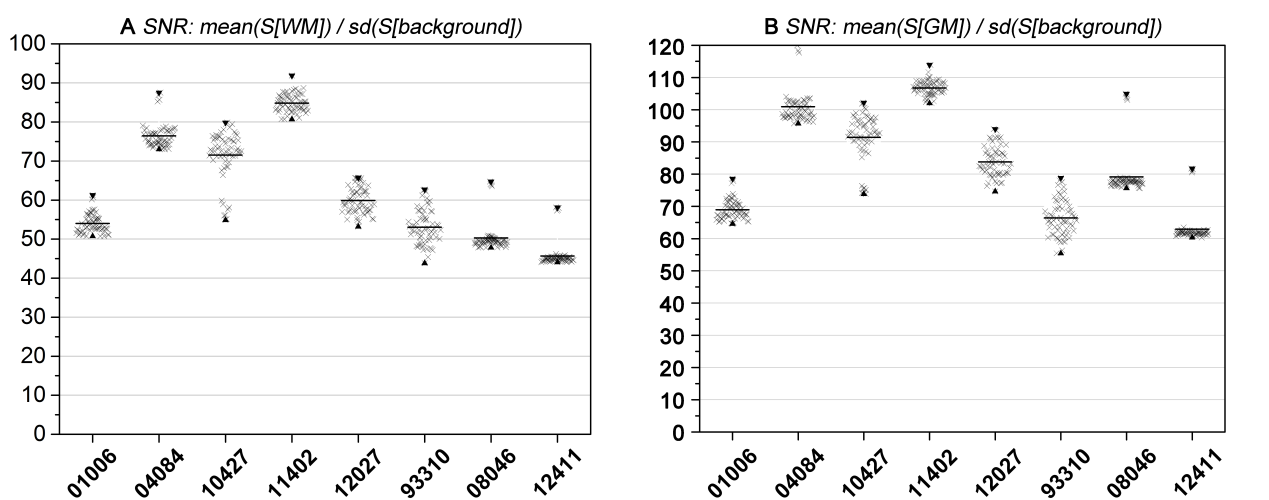
**

**Figure S1.** Scatter plot visualizing the SNRs generated by the WM (A) and the GM (B). The horizontal axis indicates 8 macaque brain specimens. ‘X’ marks showing the SNR value of the diffusion images (64 values per subject). The mean and extreme (maximum and minimum) SNRs are indicated by solid line and triangular symbols, respectively. All these SNRs are far above 20.

The primary purpose of this study was the tractography-based parcellation. Therefore, we performed a further test of the data quality by comparing the similarities between the parcellation results generated by different *b*-values. For this purpose, we prepared another macaque brain specimen using the above-mentioned operations and used it to acquire four sets of diffusion MRI data at *b*-values of 1000, 2000, 3000, and 4000 s/mm^2^ while keeping all the other parameters unchanged. The nucleus accumbens (Acb) was chosen as seeds to construct the tractography-based parcellation using these four sets of data (the individual parcellation procedure was detailed in main text). The parcellation results are shown in Figure S2; both the seeds had similar subregions distribution patterns across the MRI data for the different *b*-values (1000, 2000, 3000, and 4000 s/mm^2^), at least in 2-, 3-, 4-, 5-, and 6-cluster solutions. In particular, the 2- and 3-cluster solution results from the low *b*-value (e.g., 1000 s/mm^2^) diffusion MRI data seemed to present a high degree of overlap with those from the higher *b*-values (e.g., 4000 s/mm^2^) diffusion data, suggesting that low *b*-value diffusion data is feasible for tractography-based parcellations.

Taking above quality tests into account, the macaque diffusion MRI data acquired in this study at a relative low *b*-value of 1000 s/mm^2^ had an appropriate SNR for tractographic reconstruction and could also enable us to obtain parcellation results that are consistent with those that would have been obtained using higher *b*-values data. Thus, we concluded that the other 8 *ex vivo* macaque diffusion data should have the same ability to support the parcellation research.


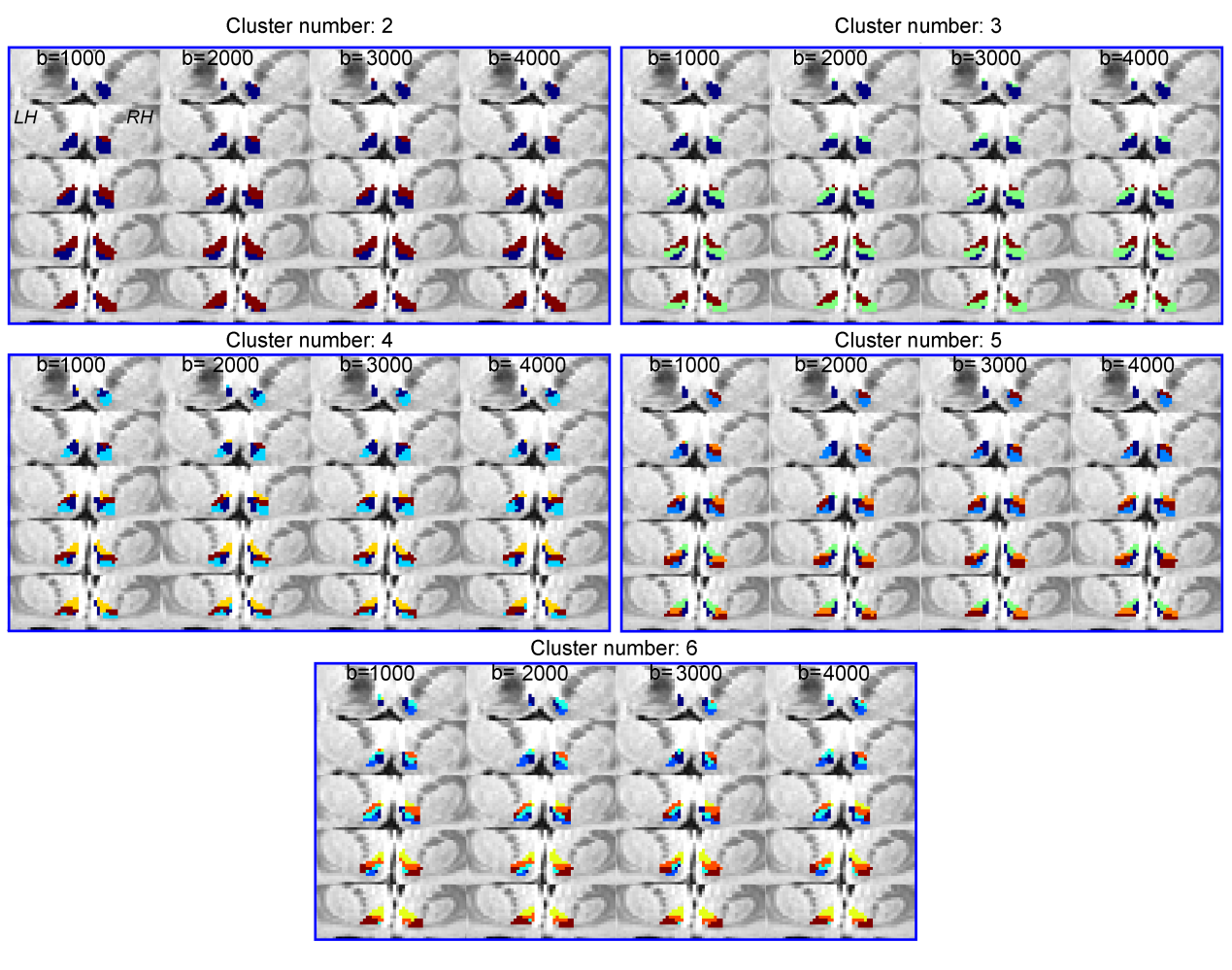
**Figure S2.** The parcellation results of the Acb. Different diffusion MRI data (*b*-values = 1000, 2000, 3000, 4000 s/mm^2^) were used for this procedure and the results are shown here for comparison. Both the two seeds were parcellated into 2, 3, 4, 5, 6 clusters. All these parcellation results show similar subregions topological distribution across datasets.

# MRI Data Preprocessing

The preprocessing of the structural images was performed as follows: correction of distortion due to magnetic field inhomogeneity, non-brain removal, calculation of the transformations between subject-native brains and a brain template (Calabrese et al., 2015) from the Montreal Neurological Institute (MNI) monkey space (Frey et al., 2011) using the symmetric normalization transformation model (Avants et al., 2008), generation the subject-native tissue maps of the gray matter (GM), white matter (WM), and cerebrospinal fluid (CSF) using the FMRIB Software Library FAST program (Zhang et al., 2001) and the prior tissue probability maps (Rohlfing et al., 2012).

The preprocessing of the diffusion images was performed as follows: correction of distortion caused by eddy currents, calculation of the transformations between subject structural and diffusion images using a 6 degrees of freedom (DOF) FMRIB's FLIRT boundary-based registration (BBR; Greve and Fischl, 2009), non-brain removal, building of the distributions of the diffusion parameters at each voxel (2 fibers per voxel; Jbabdi et al., 2012).

# Tractography-Based Parcellation Procedure

1) The seed mask defined in standard space was brought back into the subject-native structural space. After minor manual modifications of the voxels mis-registered into the WM and CSF, the mask was then brought back into subjects’ diffusion space. In subject-native diffusion space, 2) whole brain probabilistic streamline tractography was implemented for each voxel in the mask using PROBTRACKX (Behrens et al., 2007; 50,000 samples for each voxel). 3) These connectivity probability maps were thresholded at 20/50,000 as we did in earlier studies (Fan et al., 2016; Xia et al., 2017) to limit false positive connections, and then down-sampled to 2×2×2 mm for humans and to 1.2×1.2×1.3154 mm (i.e., we sampled the neighboring four voxels into one) for macaques for manageability. 4) The connection and cross-correlation matrixes (Johansen-Berg et al., 2004) were constructed in turn; and 5) the latter was fed into spectral clustering to subdivide the voxels into multiple subgroups (Baldassano et al., 2015). 6) The voxels in each subgroup were mapped back onto the brain to generate the corresponding subregion. 7) All individual parcellation results were transformed into standard space (i.e., humans: MNI space; macaques: MNI monkey space). At the group level, 8) groups of locationally corresponding subregions (one region for each individual) were extracted to generate the probability map for each subregion. 9) The maximum probability map of the seed was calculated by assigning each voxel of the reference space to the area in which it was most likely to be located (Eickhoff et al., 2006).


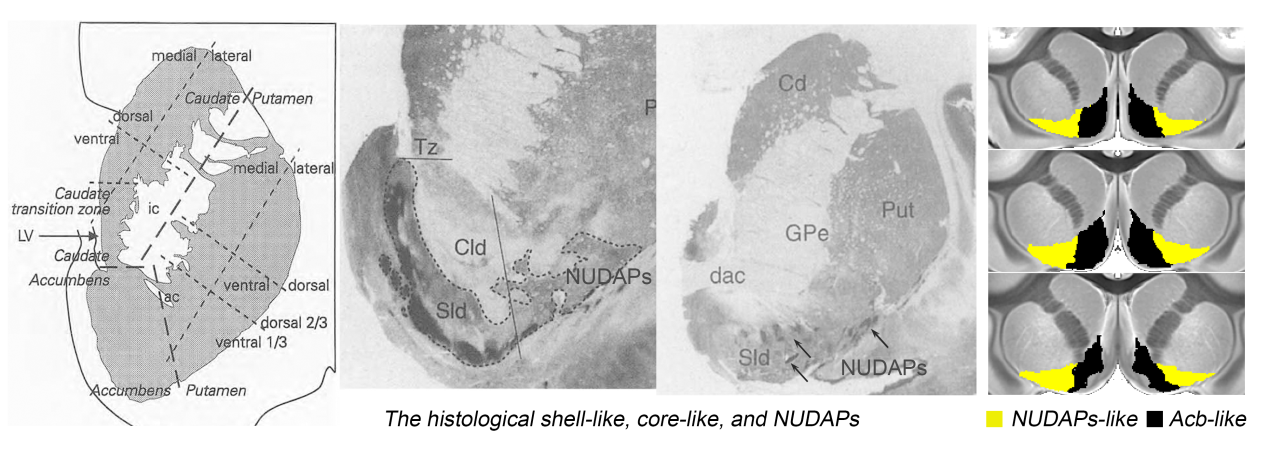


**Figure S3.** The ventral striatal subregions. The neurochemically unique domains of the accumbens and putamen (NUDAPs) in primate striatum was described as a histological region comprising many patch-like areas located in the ventral border of the Acb and the ventral one-third of the putamen ventral one-third of the putamen (left three panels with permission from Voorn et al., 1996). The solid lines in 2nd panel represent the boundaries of the Acb. Arrows in 3rd panel indicate patch-like areas (i.e., NUDAPs) in the lateral ventral striatum. This region corresponds in location with the connectivity-based division (yellow cluster in rightmost panel), and thus named as NUDAPs-like division. Acronyms: Sld, shell-like division; Cld, core-like division; Tz, transion zone; Put, putamen; dac, decussation of the anterior commissure.

# Definition of the Criteria for the Target Areas

Referring to earlier literature (Cauda et al., 2011; Xia et al., 2017), we selected areas which had strong anatomical connectivities with the ventral striatum as a member of the target group. The strong connectivity was defined according to the following criteria: 1) The number of activation voxels included in the area surpassed a fixed fraction, > 2%, of the total voxels of that area. 2) The number of activation voxels included in the area surpassed a fixed fraction, > 2%, of the total number of activation voxels.

# References

Avants, B. B., Epstein, C. L., Grossman, M., and Gee, J. C. (2008). Symmetric diffeomorphic image registration with cross-correlation: evaluating automated labeling of elderly and neurodegenerative brain. *Med. Image. Anal*. 12, 26-41. doi: 10.1016/j.media.2007.06.004

Baldassano, C., Beck, D. M., and Fei-Fei, L. (2015). Parcellating connectivity in spatial maps. *PeerJ*. 3, e784. doi: 10.7717/peerj.784

Behrens, T. E., Berg, H. J., Jbabdi, S., Rushworth, M. F., and Woolrich, M. W. (2007). Probabilistic diffusion tractography with multiple fibre orientations: What can we gain? *Neuroimage*. 34, 144-155. doi: 10.1016/j.neuroimage.2006.09.018

Budde, M. D., Janes, L., Gold, E., Turtzo, L. C., and Frank, J. A. (2011). The contribution of gliosis to diffusion tensor anisotropy and tractography following traumatic brain injury: validation in the rat using Fourier analysis of stained tissue sections. *Brain*. 134, 2248-2260. doi: 10.1093/brain/awr161

Calabrese, E., Badea, A., Coe, C. L., Lubach, G. R., Shi, Y., Styner, M. A., et al. (2015). A diffusion tensor MRI atlas of the postmortem rhesus macaque brain. *Neuroimage*. 117, 408-416. doi: 10.1016/j.neuroimage.2015.05.072

Cauda, F., Cavanna, A. E., D’Agata, F., Sacco, K., Duca, S., and Geminiani, G. C. (2011): Functional connectivity and coactivation of the nucleus accumbens: A combined functional connectivity and structure-based meta-analysis. *J. Cogn. Neurosci*. 23, 2864-2877. doi: 10.1162/jocn.2011.21624

D’Arceuil, H. E., Westmoreland, S., and de Crespigny, A. J. (2007). An approach to high resolution diffusion tensor imaging in fixed primate brain. *Neuroimage*. 35, 553-565. doi: 10.1016/j.neuroimage.2006.12.028

Dietrich, O., Raya, J. G., Reeder, S. B., Reiser, M. F., and Schoenberg, S. O. (2007). Measurement of signal-to-noise ratios in MR images: influence of multichannel coils, parallel imaging, and reconstruction filters. *J. Magn. Reson. Imaging*. 26, 375-385. doi: 10.1002/jmri.20969

Dyrby, T. B., Baare, W. F. C., Alexander, D. C., Jelsing, J., Garde, E., and Søgaard, L. V. (2011). An ex vivo imaging pipeline for producing high-quality and high-resolution diffusion-weighted imaging datasets. *Hum. Brain. Mapp*. 32, 544-563. doi: 10.1002/hbm.21043

Eickhoff, S. B., Heim, S., Zilles, K., and Amunts, K. (2006). Testing anatomically specified hypotheses in functional imaging using cytoarchitectonic maps. *Neuroimage*. 32, 570-582. doi: 10.1016/j.neuroimage.2006.04.204

Fan, L., Li, H., Zhuo, J., Zhang, Y., Wang, J., Chen, L., et al. (2016). The human brainnetome atlas: A new brain atlas based on connectional architecture. *Cereb. Cortex*. 26, 3508-3526. doi: 10.1093/cercor/bhw157

Feng, L., Jeon, T., Yu, Q., Ouyang, M., Peng, Q., Mishra, V., et al. (2017). Population-averaged macaque brain atlas with high-resolution *ex vivo* DTI integrated into in vivo space. *Brain. Struct. Funct*. 222, 4131-4147. doi: 10.1007/s00429-017-1463-6

Frey, S., Pandya, D. N., Chakravarty, M. M., Bailey, L., Petrides, M., and Collins, D. L. (2011). An MRI based average macaque monkey stereotaxic atlas and space (MNI monkey space). *Neuroimage*. 55, 1435-1442. doi: 10.1016/j.neuroimage.2011.01.040

Greve, D. N., and Fischl, B. (2009). Accurate and robust brain image alignment using boundary-based registration. *Neuroimage*. 48, 63-72. doi: 10.1016/j.neuroimage.2009.06.060

Griffanti, L., Baglio, F., Preti, M. G., Cecconi, P., Rovaris, M., Baselli, G., et al. (2012). Signal-to-noise ratio of diffusion weighted magnetic resonance imaging: Estimation methods and in vivo application to spinal cord. *Biomed. Signal. Process. Control*. 7, 285-294. doi: 10.1016/j.bspc.2011.06.003

Heimer, L. (2000). Basal forebrain in the context of schizophrenia. *Brain. Res. Brain. Res. Rev*. 31, 205-235. doi: 10.1016/S0165-0173(99)00039-9

Heimer, L., De Olmos, J. S., Alheid, G. F., Person, J., Sakamoto, N., Shinoda, K. et al. (1999). The human basal forebrain. Part II. Bloom F.E., Bjorkland A., Hokfelt T. Amsterdam: Elsevier; Handbook of Chemical Neuroanatomy. 57-226.

Jbabdi, S., Sotiropoulos, S. N., Savio, A. M., Graña, M., and Behrens, T. E. (2012). Model-based analysis of multishell diffusion MR data for tractography: how to get over fitting problems *Magn. Reson. Med*. 68, 1846-1855. doi: 10.1002/mrm.24204

Johansen-Berg, H., Behrens, T. E., Robson, M. D., Drobnjak, I., Rushworth, M. F., Brady, J. M. et al. (2004). Changes in connectivity profiles define functionally distinct regions in human medial frontal cortex. *Proc. Natl. Acad. Sci. USA*. 101, 13335-13340. doi: 10.1073/pnas.0403743101

Mukherjee, P., Chung, S. W., Berman, J. I., Hess, C. P., and Henry, R. G. (2008). Diffusion Tensor MR Imaging and Fiber Tractography: Technical Considerations. *AJNR. Am. J. Neuroradiol*. 29, 843-852. doi: 10.3174/ajnr.A1052

Neto, L. L., Oliveira, E., Correia, F., and Ferreira, A. G. (2008). The human nucleus accumbens: where is it? A stereotactic, anatomical and magnetic resonance imaging study. *Neuromodulation*. 11, 13-22. doi: 10.1111/j.1525-1403.2007.00138.x

Rane, S., Nair, G., and Duong, T. Q. (2010). DTI at long diffusion time improves fiber tracking. *NMR. Biomed*. 23, 459-465. doi: 10.1002/nbm.1482

Rohlfing, K. J., Longo, M. R., and Bertenthal, B. I. (2012). Dynamic pointing triggers shifts of visual attention in young infants. *Dev. Sci*. 15, 426-435. doi: 10.1111/j.1467-7687.2012.01139.x

Salgado, S., and Kaplitt, M. G. (2015). The nucleus accumbens: A comprehensive review. *Stereotact. Funct. Neurosurg*. 93, 75-93. doi: 10.1159/000368279

Voorn, P., Brady, L. S., Berendse, H. W., and Richfield, E. K. (1996). Densitometrical analysis of opioid receptor ligand binding in the human striatum – I. Distribution of u opioid receptor defines shell and core of the ventral striatum. *Neuroscience*. 75, 777-792. doi: 10.1016/0306-4522(96)00271-0

Xia, X., Fan, L., Cheng, C., Eickhoff, S. B., Chen, J., Li, H. et al. (2017). Multimodal Connectivity-Based Parcellation Reveals a Shell-Core Dichotomy of the Human Nucleus Accumbens. *Hum. Brain. Mapp*. 38, 3878-3898. doi: 10.1002/hbm.23636

Zhang, Y., Brady, M., and Smith, S. (2001). Segmentation of brain MR images through a hidden Markov random field model and the expectation-maximization algorithm. *IEEE. Trans. Med*. *Imaging*. 20, 45-57. doi: 10.1109/42.906424
